# Philadelphia Hospital

**Published:** 1859-10

**Authors:** 


The new Medical Board of the Philadelphia Hospital has assumed the control of the institution. Rules for its government in accordance with the present arrangement, have been adopted, which will enable its affairs to be conducted in the orderly and systematic manner so essential to the harmony and efficiency of a large hospital. The general organization is similar to that of the Pennsylvania Hospital, and most of the other prominent institutions of the kind in this country.

Dr. S. D. Gross has been elected President of the Medical Board.

The Philadelphia Hospital is the largest hospital on this continent, presenting an unequaled variety of disease, and has no superior in its opportunities for clinical study.

